# Harmonizing platelet function analyzer testing and reporting in a large laboratory network

**DOI:** 10.1111/ijlh.13907

**Published:** 2022-06-26

**Authors:** Emmanuel J. Favaloro, Soma Mohammed, Ronny Vong, Kent Chapman, Geoffrey Kershaw, Sarah Just, Lynne Connelly, Michael Ryan, Diane Zebeljan, Timothy Brighton, Leonardo Pasalic

**Affiliations:** ^1^ Haematology, Institute of Clinical Pathology and Medical Research (ICPMR), NSW Health Pathology, Westmead Hospital Westmead New South Wales Australia; ^2^ Sydney Centres for Thrombosis and Haemostasis Westmead New South Wales Australia; ^3^ Faculty of Science and Health Charles Sturt University Wagga Wagga New South Wales Australia; ^4^ Haematology, NSW Health Pathology John Hunter Hospital Newcastle New South Wales Australia; ^5^ Haematology, NSW Health Pathology Prince Alfred Hospital Camperdown New South Wales Australia; ^6^ Haematology, NSW Health Pathology Royal North Shore Hospital St Leonards New South Wales Australia; ^7^ Haematology, NSW Health Pathology Wollongong Hospital Wollongong New South Wales Australia; ^8^ Haematology, NSW Health Pathology Liverpool Hospital Liverpool New South Wales Australia; ^9^ Haematology, NSW Health Pathology Prince of Wales Hospital Randwick New South Wales Australia; ^10^ Westmead Clinical School University of Sydney Westmead New South Wales Australia

**Keywords:** harmonization, PFA‐100, PFA‐200, platelet function analyzer

## Abstract

**Introduction:**

The platelet function analyzer (PFA) is a popular platelet function screening instrument, highly sensitive to von Willebrand disease (VWD) and to aspirin therapy, with moderate sensitivity to defects in platelet function and/or deficiencies in platelet number. There are two models, the original PFA‐100 and the contemporary PFA‐200. Normal reference ranges (NRRs) provided by the manufacturer are the same for both models, instead being based on the type of test cartridge, for which there are two main ones: collagen/epinephrine (C/Epi) and collagen/adenosine diphosphate (C/ADP).

**Methods:**

Comparative evaluations of PFA testing and reporting in six different sites of a large pathology network, aiming to harmonize NRRs and test reporting across all network sites. A separate comparative study of testing a range of samples (*n* > 150) on a PFA‐100 versus that on a PFA‐200. Review of contemporary literature.

**Results:**

Each site was identified to have a different reporting NRR, which after consolidating data permitted establishment of an agreed harmonized NRR for use across the network (C/Epi: 90–160; C/ADP: 70–124; based on *n* > 180). Similarly, each site reported and interpreted results in different ways, and after discussion and consolidation, a harmonized approach to interpretation and reporting was achieved. The separate comparative study of PFA‐100 versus PFA‐200 testing confirmed instrument equivalence.

**Conclusion:**

We achieved harmonized NRRs and reporting for PFA testing across a large pathology network. Our approach may be useful for other laboratory networks wishing to harmonize PFA testing.

## 1 INTRODUCTION

The platelet function analyzer (PFA) is a popular platelet function screening instrument, originally described over 25 years ago, in 1995.^1^, ^2^ The PFA was based on the technology of an earlier instrument, the Thrombostat 4000, which was otherwise identified as an “in vitro bleeding time.”^3^ The originally released model of the PFA was called the PFA‐100, and likewise became known as an “in vitro bleeding time.”^4^, ^5^ The PFA‐100 is widely distributed internationally, including Europe, Asia Pacific, and North America. A newer model of the PFA, called the PFA‐200, has been released in some countries, but this currently excludes the United States. Nevertheless, the PFA‐200 will become the dominant instrument over time, as the manufacturer (Siemens Healthineers) has an active program of replacing the existing PFA‐100 intruments with their newer PFA‐200, and ceasing service contracts with the PFA‐100, at least in Australia.

The PFA‐100 and PFA‐200 both report test results as a “closure time” (CT), which is the time (in seconds, s) in which an aperture in the test cartridge is blocked, after whole blood is introduced into the cartridge, flows through the cartridge under sheer pressure, and finally makes contact with the test membrane coated with the various platelet agonists. Platelets adhere to the membrane, then become activated and aggregate, eventually blocking the aperture. There are three different test cartridge types, according to the platelet agonists coated onto a membrane within the cartridge, and namely collagen/epinephrine (C/Epi), collagen/adenosine diphosphate (C/ADP), and the so‐called Innovance PFA P2Y cartridge coated with ADP, prostaglandin E1 and calcium chloride. Like the PFA‐200, the Innovance PFA P2Y, marketed as sensitive to P2Y12 antagonist therapy, such as clopidogrel, is not available in all geographies, including the United States. Moreover, the C/Epi and C/ADP test cartridges are those primarily used within most laboratories, and are the subject of the current report.

The PFA is sensitive to disturbances in the primary haemostasis, as well to a variety of drugs, supplements and foods.^6^, ^7^, ^8^, ^9^, ^10^ In particular, the PFA is highly sensitive to deficiency or defect in von Willebrand factor (VWF), and thus to the presence of von Willebrand disease (VWD).^6^, ^7^, ^8^, ^9^, ^10^ Indeed, a normal PFA test result using the C/Epi test cartridge represents a highly effective negative exclusion for VWD, in some laboratories even better than individual tests for VWF level and activity.^6^, ^7^, ^8^ The PFA is also highly sensitive to aspirin anti‐platelet therapy, in particular using the C/Epi test cartridge,^9^, ^10^ but only moderately sensitive to deficiency of platelets or the presence of platelet dysfunction.^6^, ^9^, ^10^ The PFA is also sensitive to a plethora of other drugs and supplements, as well as certain foods (including high garlic, chocolate or fish oil intake).^10^ The PFA is also sensitive to haematocrit levels.^10^ Overall, then, prolongation in PFA CTs may reflect one or more of a variety of events, and thus is not specific for any particular defect, and normal CTs, for example to exclude VWD, seem to have better clinical utility.

Notably, the results of testing via the PFA‐100 and the PFA‐200 are considered “identical,” in so far as the manufacturer provided CT normal reference ranges (NRRs) for the two instrument platforms are identical. That is, the CT NRRs are not instrument‐centric, but rather are based on the test cartridge type used by the instruments. The manufacturer CT NRRs for the C/Epi and C/ADP test cartridges are, respectively, 82–150 and 62–100 s, for use on both the PFA‐100 and PFA‐200. However, the previous extensive review of the literature shows an extraordinary range of reported NRRs in actual use in different laboratories.^10^ Indeed, we confirmed the use of different NRRs at each site in which a PFA‐100/200 was in use even in our laboratory network, NSW Health Pathology (NSWHP). Moreover, each site in our network interpreted and reported differently in terms of associated test result comments for requesting clinicians. The current study therefore reports on our harmonization of CT NRRs and test reporting for PFA‐100/200 for all sites in NSWHP.

## 2 MATERIALS AND METHODS

### 2.1 Overview of setting and study design

This evaluation was undertaken by NSWHP personnel, and intended to achieve harmonization of PFA‐100/200 NRRs and reporting in all NSWHP laboratories performing PFA tests. In Australia, PFA testing is used by laboratories as a screen of platelet function and VWD. Such testing is regulated by several Australian agencies.^11^ First, laboratory instruments and reagents (“in vitro diagnostics”; IVDs) require regulatory approval by the Therapeutic Goods Administration (TGA). Laboratory testing practice also requires accreditation status, which is effected by the National Association of Testing Authorities (NATA), in part using guidance from the National Pathology Accreditation Advisory Council (NPAAC). In relation to PFA testing, laboratories are required to establish or verify NRRs for CTs for use. The simplest approach is verification, which can employ straightforward methods as per Clinical and Laboratory Standards Institute (CLSI) guidance.^12^ In addition, there is a requirement for ongoing quality control and participation in external quality assessment (EQA), which in Australia is undertaken by the Royal College of Pathologists of Australasia Quality Assurance Program (RCPAQAP).^13^, ^14^, ^15^

Given PFA testing is only performed within the larger sites of our organization, six major sites (Supplementary Table S1) partook in this study. Specifically, each site provided historical data for normal individual samples assessed at each site as part of their internal validation studies for their instrument installation, and as potentially used to derive local CT NRRs. Subsequent to historical PFA installation, all sites have been accredited for PFA testing, and participate in ongoing EQA for these tests. The historical data were assessed separately and in composite to eventually establish a harmonized NRR for the entire network. The second process was undertaken to establish harmonized reporting of associated interpretive comments, which are used to explain PFA test results to requesting clinicians. This was undertaken by discussion and agreement of lead scientists and haematologists. The third evaluation was undertaken at the Institute of Clinical Pathology and Medical Research (ICPMR) site to confirm comparability of PFA‐100 and PFA‐200 instrumentation. In this substudy, approximately 20–30 samples (representing a variety of CT values; both normal and prolonged) were co‐tested per year from 2014 (when the PFA‐200 was acquired) onward, as part of a quality control process to assess and confirm ongoing equivalence for patient reporting purposes (laboratory accreditation requirement). This process is summarized in Supplementary Figure S1.

The instruments in place at the six sites comprised both PFA‐100 and PFA‐200 instruments (Supplementary Table S1). All samples utilized were based on sodium citrate (3.2%) anticoagulation, employing whole blood collected and processed according to the PFA manufacturer instructions, with testing completed within 4 h of blood collection.

### 2.2 Verification/establishment of CT NRRs and Statistical analysis

We performed several procedures to establish, or verify manufacturer recommended, CT NRRs for both C/Epi and C/ADP test cartridges, in part utilizing the CLSI guidance document “Defining, Establishing, and Verifying Reference Intervals in the Clinical Laboratory”.^12^ This study primarily used historical data as collected and utilized at each site at implementation of PFA testing at that site, as well as normal individual data potentially collected since implementation at some sites. The normal individuals most typically comprised laboratory or other staff from our organization. Both male and female adults (>18 yr) were included, and as there is no gender or adult age‐related differences in PFA CTs, gender and age was not always documented. All testing was performed within 4 h of sample collection. Although we attempted to exclude individuals who were on antiplatelet medication, this was assessed inconsistently between sites, and it is possible that some individuals had recently consumed undisclosed agents capable of affecting platelet function (e.g., agents used to treat headaches and other aches and pains; supplements or foods). Such undisclosed intake may have led to occasional high‐outlier CT data. Not all sites assessed these collected samples for platelet count, haematocrit, and/or VWF level and activity, all of which are also known to affect PFA CTs. However, data from several sites where this did occur is available and confirmed normal platelet count, normal haematocrit, and normal levels of VWF for these data subsets in almost all cases. Nevertheless, an undetected low platelet count, haematocrit of VWF level or activity may occasionally have also yielded high‐outlier CT data. We assessed agreement (or lack thereof) of NRRs between individual sites, and also evaluated site data as well as composite data for normality by several statistical methods using the GraphPad Prism software (La Jolla, CA, USA). The four normality tests available in this software are the Anderson–Darling, D'Agostino and Pearson, Shapiro–Wilk and Kolmogorov–Smirnov tests. We also calculated normal ranges based on various procedures, ultimately deciding on 2.5–97.5th percentiles, because the arising data did not consistently show a normal distribution. We also ultimately decided on 2.5–97.5th percentiles for composite data after the removal of visual outliers. Comparative data using separate processes of statistical outlier removal was also performed (using the Prism recommended Rout test, and a common Tukey inter‐quarter range‐based test) and results of these are shown for comparison. Data are otherwise presented numerically or in a qualitative synthesis.

### 2.3 Ethical considerations

According to the guidance from local Human Research Ethics Committees, formal ethical approval for this study was not sought, as the evaluation represents a Quality Assurance project of method verification.

### 2.4 Literature search

A PubMed search of ((PFA 100) or (PFA 200) or “PFA”) was also undertaken (on March 10, 2022) without time restriction to primarily identify contemporary literature, in particular those published studies reporting PFA‐100/200 CT NRRs. The search generated 1202 reports. We selected the most recent 100 of these reports in English language, and thus representing studies conducted in the last 3 years, and searched these to identify any study reporting a NRR for either C/Epi and/or C/ADP. This permitted identification of what we felt was a selection of the most recent “contemporary” NRRs reported in the literature. This “contemporary” dataset was compared to NRRs previously identified in an earlier literature review^10^ as used to identify a “historical” dataset. Furthermore, the PubMed search generating 1202 reports was additionally searched by adding the word “harmoni*” to attempt to capture any relevant articles reflecting harmonization (or harmonization) of PFA CTs. This resulted in the capture of two papers, neither of which discussed harmonization of PFA CTs.

## 3 RESULTS

### 3.1 Individual site and composite data for NRR determination

**FIGURE 1:**
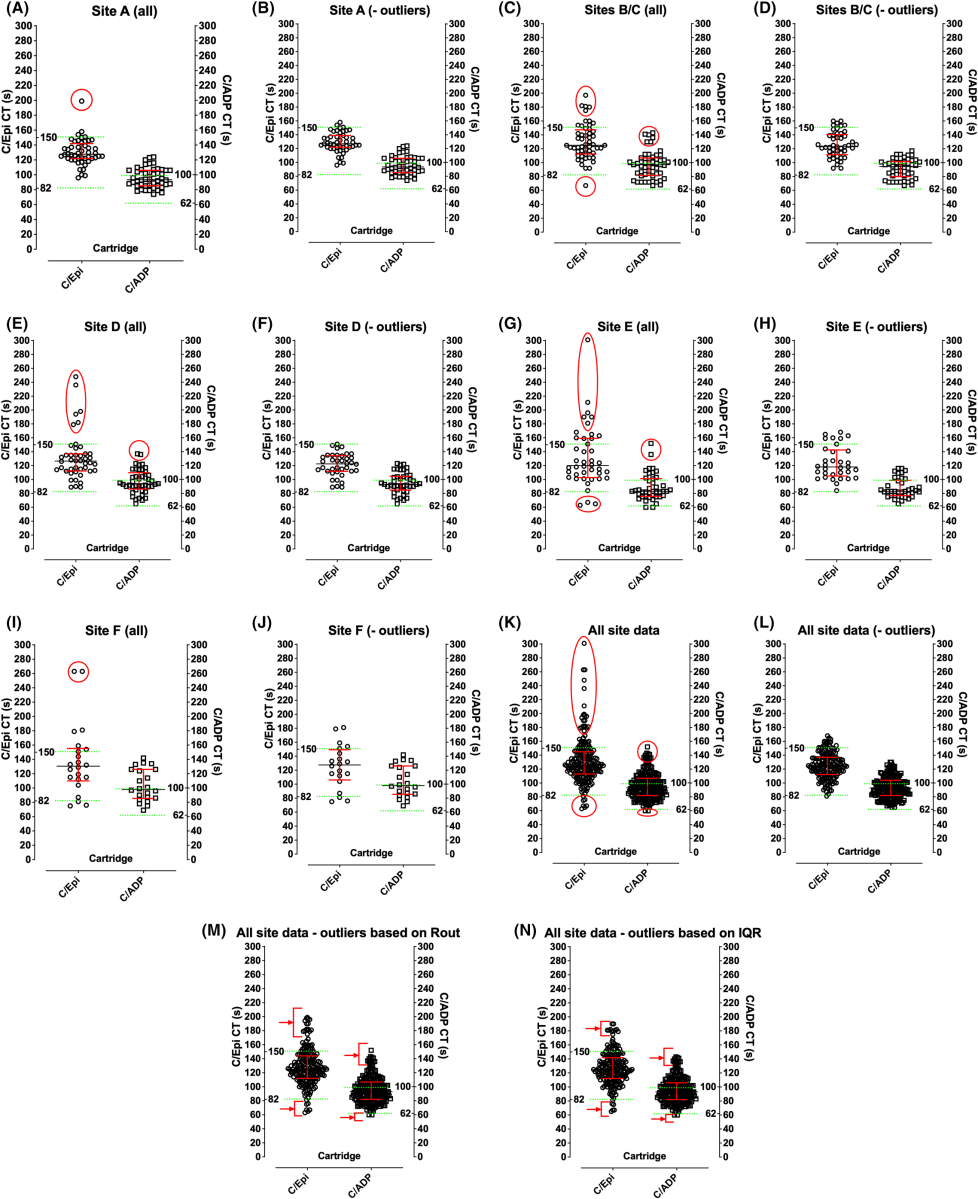
Normal reference range data sets from each site plus composite data. Figures A, C, E, G, and I show individual site data with visual outliers identified in red circles. Figures B, D, F, H, and J show individual site data with visual outliers removed. Figures K and L show composite data with visual outliers identified (K) and removed (L). Figures M and N show composite data with statistical outliers removed according to two separate procedures; some visual outliers are still evident in these (arrowed). Left *y*‐axis in each figure shows C/Epi CT in seconds; right y‐axis shows C/ADP CT in seconds. The Siemens product information CT NRRs are shown by the green horizontal dashed lines.

Individual site data are shown in Figure 1, and summarized in Table 1. Each site performed testing using between 22–48 individual normal samples. Some data points could be visually observed to be clear outlier points (identified in Figure 1 using red circles), with some sites showing a greater proportion of visual outliers. These outliers could represent undisclosed use of an anti‐platelet agent or other compound/supplement affecting PFA CTs, or unrecognized low platelet counts, haematocrits or VWF. However, where data was available, platelet counts, haematocrits and VWF level and activity were generally normal for samples collected from these normal individuals (Supplementary Figure S3). Also, there was limited correlation between PFA CTs and platelet count or haematocrit for this normal data set; however, there was some correlation with VWF level and activity, especially for C/ADP (Supplementary Figure S3).

**TABLE 1:** Individual site and composite data for NRR determination

Data set from site	No. donors Col/ADP, Col/Epi	Col/ADP	Col/Epi
Original Manufacturer NRR (PFA‐100 and PFA‐200)	309	62–100	82–150
*Current NRRs*			
A. ICPMR		64–127	94–162
B/C. JHH/RNSH (harmonized; manufacturer)		62–100	82–150
C. RNSH (historical; pre‐harmonization)		73–127	94–162
D/E. RPA/Liverpool (harmonized)		60–120	80–170
E. Liverpool (historical; pre‐harmonization)		73–127	81–146
F. ISLHD		71–125	94–193
*Calculated NRRs (− visual outliers; 2.5th–97.5th percentiles)*			
A. ICPMR	48, 47	75–124	97–157
B/C. JHH/RNSH	46, 46	67–116	92–160
D. RPA	42, 38	65–123	89–151
E. Liverpool	38, 33	65–116	84–168
F. ISLHD	22, 20	69–142	75–181
**Composite NSWHP NRR (− visual outliers)**	194, 184	69–124	89–160
**Composite NSWHP NRR (− statistical outliers** ^**a**^ **)**	208, 202	**67–139**	**75–194**
**Composite NSWHP NRR (− statistical outliers** ^**b**^ **)**	207, 196	**67–137**	**76–181**
**Composite NSWHP NRR (− visual outliers) (minor rounding). Ranges to be adopted for harmonization**	194, 184	**70–124**	**90–160**

^a^ Using the Prism recommended Rout method and selecting 5% as Q. Selecting 10% as Q did not exclude any more outliers; selecting 2% as Q resulted in 1 less outlier for C/Epi.

^b^ Using the Tukey interquartile range (IQR) method.

**FIGURE 2:**
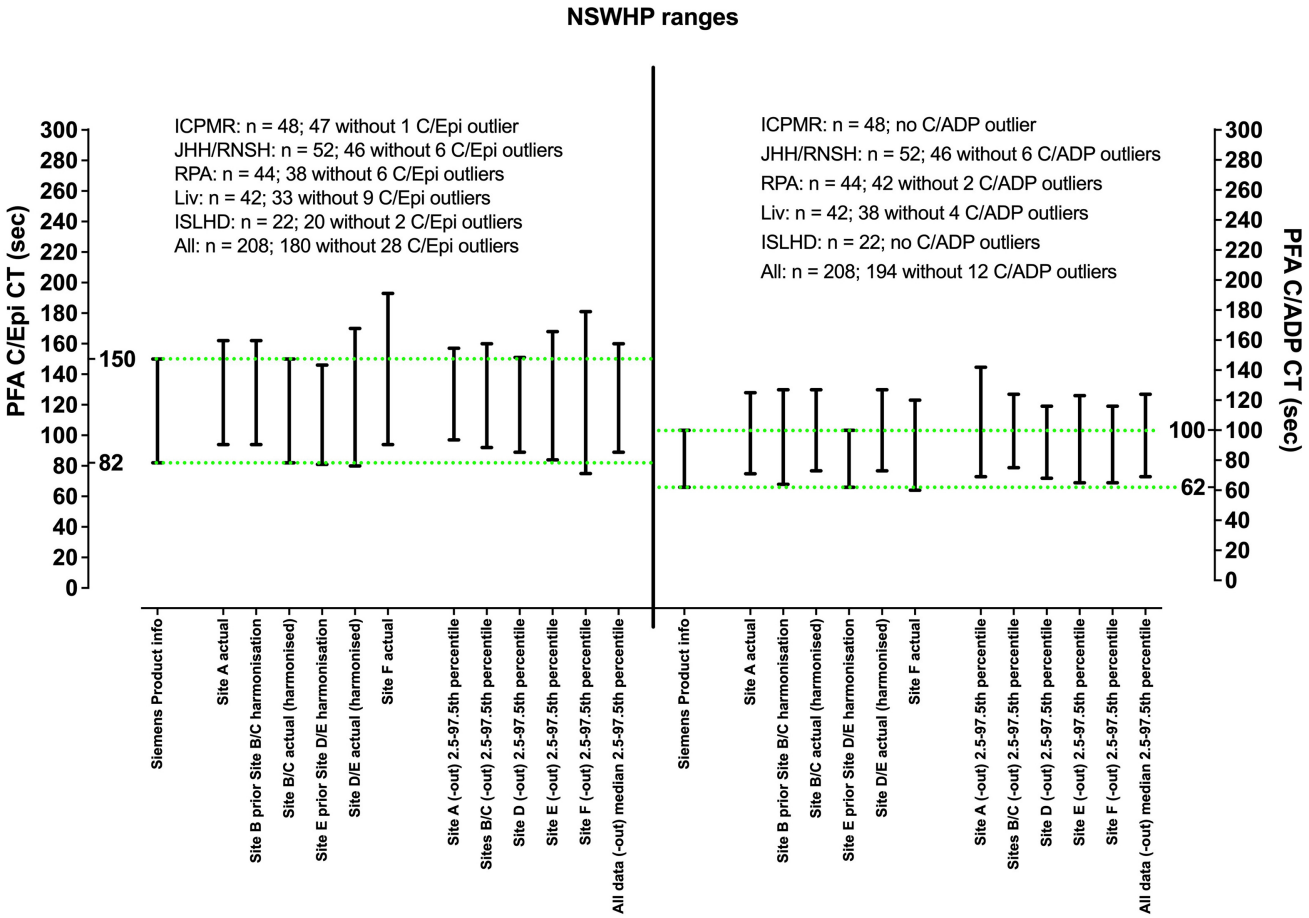
Normal reference ranges calculated from data sets in Figure 1 (with outliers removed), as well as those active prior to harmonization. Left *y*‐axis shows C/Epi CT in seconds; right *y*‐axis shows C/ADP CT in seconds. The Siemens product information CT NRRs are shown by the green horizontal dashed lines.

Removal of visual outlier data points was undertaken to calculate “site‐specific” and overall composite NRRs. Using CLSI guidance,^12^ manufacturer recommended CT NRRs for C/Epi could theoretically be adopted at some sites, with >90% of data points within the manufacturer ranges, but not at other sites (where <90% data points within the manufacturer ranges). In general, the manufacturer recommended ranges could not be adopted for C/ADP at any site, with <90% data points within the manufacturer ranges. Interestingly, data passed several tests of normality for some sites, but not at other sites (Supplementary Table S2). Data from all sites were combined, as also shown in Figure 1, and summarized in Table 1. Again, visual outlier data points, potentially representing undisclosed anti‐platelet agents/supplements or low platelet count/haematocrit/VWF were removed as a comparative exercise. Composite data largely agreed with the manufacturer recommended range for C/Epi, but not for C/ADP. This composite data passed tests for normality for C/Epi, but not for C/ADP (Supplementary Table S2). Accordingly, it was decided to calculate NRRs based on 2.5–97.5th percentiles, as shown in Figure 2 and Table 1. Table 1 also shows NRRs that were currently in place at each site prior to this study. Notably, several sites had already “harmonized” NRRs with each other (Table 1). Nevertheless, the NRRs in use at different sites, or otherwise calculated based on 2.5–97.5th percentiles, differed from one another. In addition to the removal of visual outliers, composite data were also evaluated for outlier removal using several statistical tests, as shown in Figure 1 and Table 1. These statistical tests removed far fewer outliers than those visually identified, with some residual CTs still appearing as visual outliers (Figure 1). The final composite ranges agreed by the team are also shown in Table 1, these being 70–124 (C/ADP) and 90–160 (C/Epi).

### 3.2 Literature‐reported NRRs

**FIGURE 3:**
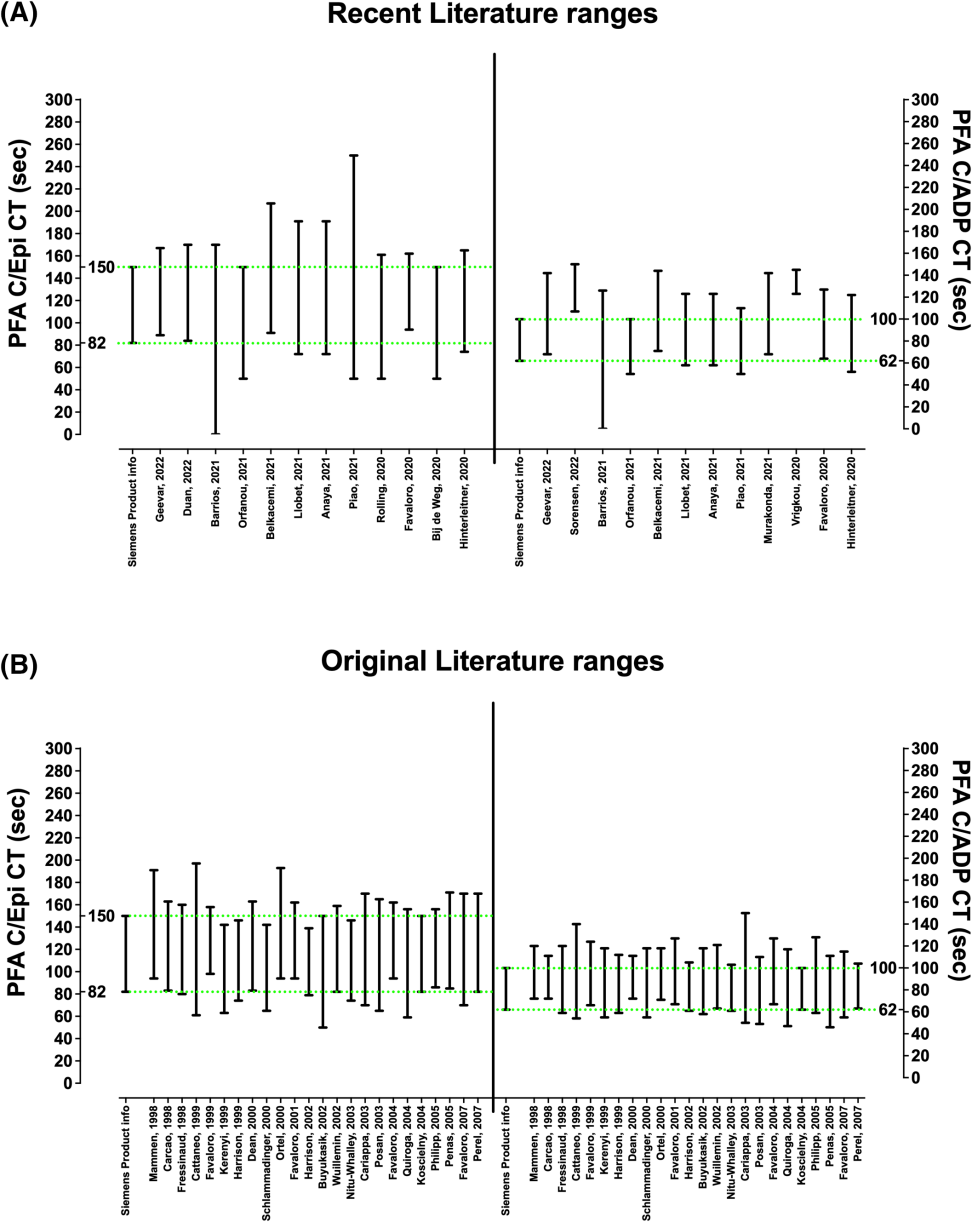
A selection of CT NRRs from published literature. Left y‐axes show C/Epi CT in seconds; right *y*‐axes show C/ADP CT in seconds. The Siemens product information CT NRRs are shown by the green horizontal dashed lines. Figure A shows NRRs from some contemporary literature as obtained from the recent literature search (see Methods), and reflecting information from the past 3 years.^6^, ^16^, ^17^, ^18^, ^19^, ^20^, ^21^, ^22^, ^23^, ^24^, ^25^, ^26^, ^27^, ^28^, ^29^ Figure B shows NRRs from some early literature as otherwise reviewed in Reference 10.^30^, ^31^, ^32^, ^33^, ^34^, ^35^, ^36^, ^37^, ^38^, ^39^, ^40^, ^41^, ^42^, ^43^, ^44^, ^45^, ^46^, ^47^, ^48^, ^49^, ^50^, ^51^, ^52^, ^53^ Reference numbers identify their place in the reference list. In recent literature, many publications only provide an upper cut‐off value, since they were only interested in CT prolongations. These data have arbitrarily been given a lower limit value of 50s to enable a range to be shown. One publication (Reference 18) indicated NRRs for both C/Epi and C/ADP starting at 0, which is not feasible.

For comparison, some CT NRRs reported in the contemporary^6^, ^16^, ^17^, ^18^, ^19^, ^20^, ^21^, ^22^, ^23^, ^24^, ^25^, ^26^, ^27^, ^28^, ^29^ and original literature^30^, ^31^, ^32^, ^33^, ^34^, ^35^, ^36^, ^37^, ^38^, ^39^, ^40^, ^41^, ^42^, ^43^, ^44^, ^45^, ^46^, ^47^, ^48^, ^49^, ^50^, ^51^, ^52^, ^53^ are shown in Figure 3. In most instances, these NRRs differ from each other and also from the manufacturer ranges, especially for C/ADP.

### 3.3 PFA‐100 versus PFA‐200 testing

One site, the ICPMR, had both a PFA‐100 and PFA‐200 on site, and had performed parallel testing over several years (since 2014) in order to show ongoing comparability as part of a quality control process. This data is summarized in Supplementary Figure S2, and largely confirms that CTs from these instruments were comparable.

### 3.4 Interpretive reporting

**FIGURE 4:**
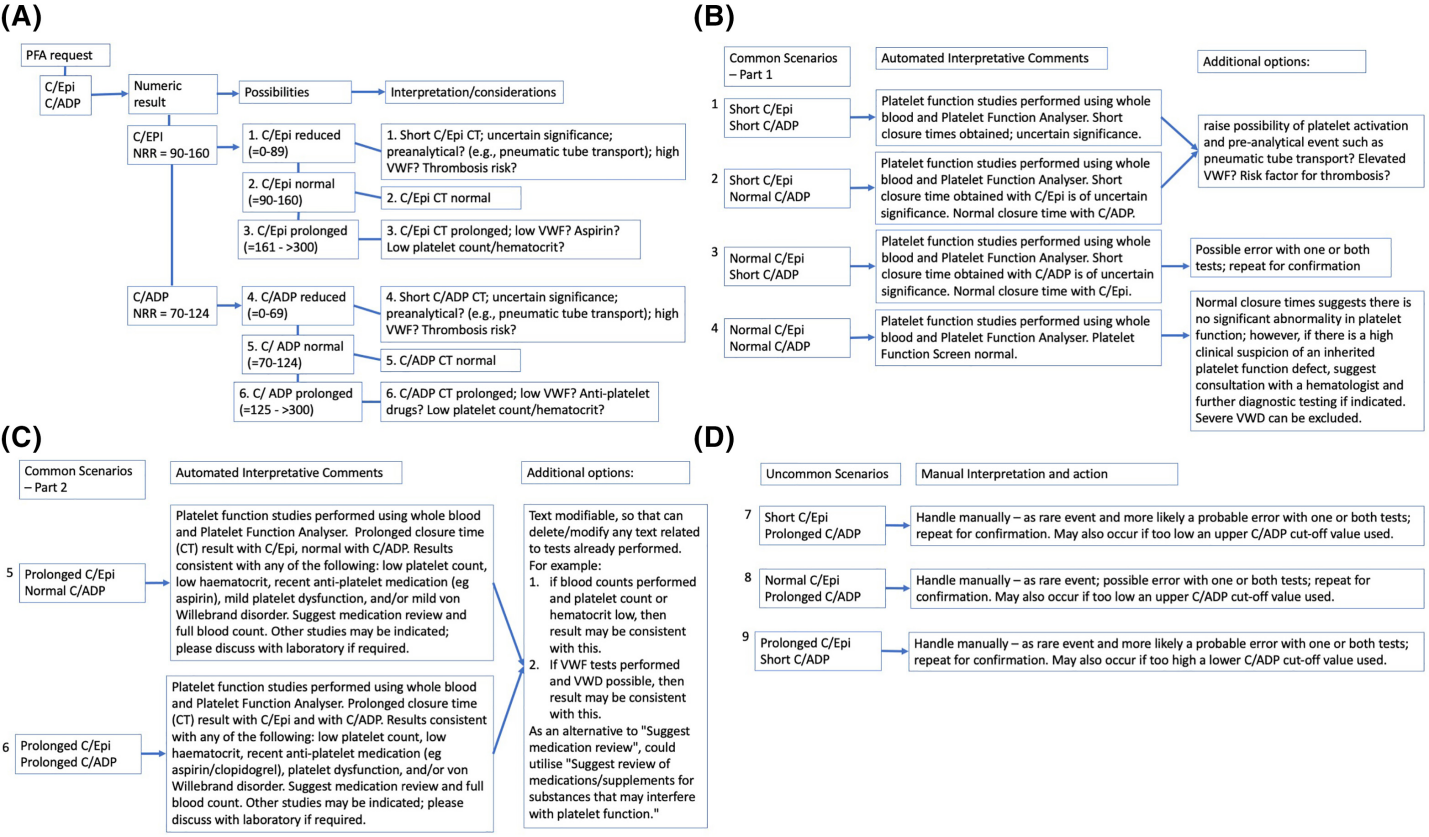
NSWHP harmonized interpretive comments to accompany numeric test results. Figure A shows an algorithm with representative strategy according to different groups of data for C/Epi and C/ADP CTs. In total, with three possible outcomes for C/Epi (short, normal or prolonged CT) and for C/ADP, nine different scenarios are possible. Figure B shows four common scenarios for combining short and normal C/Epi and C/ADP CT data, together with the harmonized automated interpretative comments, plus additional options for investigation. Figure C shows two common scenarios for combining normal and prolonged C/Epi and C/ADP CT data, together with the harmonized automated interpretative comments. Figure D shows three uncommon scenarios combining unexpected C/Epi and C/ADP CT test patterns, together with manual interpretation and suggested follow‐up action

After discussion and agreement, the final harmonized interpretive comments as associated to PFA test results, and for the benefit of requesting clinicians, is shown in Figure 4. Discussion and agreement involved scientific representatives of all participant sites, as well as lead clinical haematologists/pathologists. In brief, there are three possibilities for each cartridge, being below, within, and above the NRR limits. Thus, using both C/Epi and C/ADP test cartridges, there are 3 × 3 (=9) possible scenarios, with some scenarios being more common than others, and some scenarios being rare.

## 4 DISCUSSION

To our knowledge, this is the only report of multi‐site harmonized PFA CT NRRs and test interpretation/reporting in the literature. The harmonized C/Epi NRR is similar to the manufacturer recommended NRR, but the harmonized C/ADP NRR is quite different (essentially much wider). A similar picture is actually observed in the literature, with C/Epi NRRs being more similar to manufacturer ranges than C/ADP (Figure 3). However, what is more striking is the dissimilarity in NRRs in use from laboratory‐to‐laboratory (Figure 3), including within the same laboratory network (Table 1). To some extent, this may be due to the use of small groups of normal individuals for generation of “less accurate” NRRs (see, e.g., different NRRs calculated for each site (Table 1, Figure 2). Moreover, high‐outlier CTs could potentially be due to undisclosed antiplatelet agents or other compounds/supplements known to affect PFA CTs, as well unrecognized low platelet counts/haematocrits/VWF levels, and low‐outlier CTs potentially due to high levels of VWF or inflammation.^9^, ^10^ In some cases, from the literature, this may be compounded by the statistical method used to generate NRRs, be it +/− 2 standard deviations (SDs) as is often done, 5‐95th percentiles, or 2.5–97.5th percentiles as in our case. Interestingly, most papers in the literature do not disclose the number of normal individuals used to generate NRRs, nor the statistical method used. Critically, the “correct” upper cut‐off value is important to optimize detection or exclusion of primary haemostasis defects, such as VWD. As an example, the ICPMR laboratory recently published its extensive validation of the PFA in terms of excluding VWD should normal PFA CTs be found.^6^ In this study, the ICPMR NRRs of 64–127 (C/ADP) and 94–162 (C/Epi) were employed, thereby “validating” these NRRs for such purpose. An earlier but the similar study from France showed the similar utility for the PFA in VWD exclusion.^8^

Notably, too high a CT cut‐off, especially for C/Epi, may miss cases of primary haemostasis defects, and too low a cut‐off may over‐call the presence of primary haemostasis defects. Many papers from the literature only cite a high‐level cut‐off CT value, since this is seen as important for recognition of bleeding risk, due to VWD, platelet dysfunction, low platelet count, anti‐platelet medication (etc). Although low‐level cut‐off values seem to have lower utility, especially for identification/exclusion of primary haemostasis defects, they may identify other pertinent issues, such as pre‐analytical problems (e.g., platelet activation due to pneumatic tube transport often results in short CTs). Moreover, short CTs have also been associated with increased thrombotic risk, in part reflective of high levels of VWF activity.^6^, ^54^ Thus, we feel it important to identify the full range to include both lower and upper thresholds.

We chose to use visual identification of outliers for removal, and as potentially reflective of undisclosed or unrecognized confounders, and for which there are many possibilities.^9^, ^10^ In particular, aspirin and non‐steroid anti‐inflammatory agents such as ibuprofen are in widespread use in a range of compounds, including for acute analgesic pain relief. Moreover, use of statistical tools to assess outlier removal identified much fewer outliers and generate much wider NRRs (Table 1), which reflected even higher discordance to both manufacturer ranges and most literature data. Finally, visual outlier removal resulted in NRRs that were close to that verified as clinically useful for VWD exclusion,^6^ whereas statistical outlier removal retained many data points that visually seemed to be outliers, resulting in much wider NRRs (Table 1). As there is no gold standard process for outlier removal, and as visual outlier removal remains a tool used by many in haemostasis, we feel that this provided the best outcome in our study.

Our study also confirmed the essential equivalence of CTs from PFA‐100 or PFA‐200 (Supplementary Figure S2). We are not aware of any similar study in the literature. Importantly, the equivalence of PFA‐100 and PFA‐200 CTs has also been shown using EQA data.^13^, ^14^, ^15^ Regarding the interpretive comments, we hope that these are comprehensive enough to enable laboratories to choose the best options for their own diagnostic practice.

We acknowledge several limitations in our study. This study was a laboratory‐based evaluation, and did not further assess the “utility” of these ranges to identify or exclude primary haemostasis defects, based on clinical criteria. Further, we did not specifically assess the influence of all variables, such as platelet count or haematocrit, on PFA test results, and undisclosed antiplatelet compounds could have led to high CTs in the NRR data set. One the other hand, the ICPMR lab has published some clinical utility data,^6^ and using similar NRRs to those ultimately adopted by the group.^6^ As a strength, our composite study data comprised in excess of 180 normal individuals, and is likely one of the larger datasets used by laboratories attempting to establish CT NRRs for PFA testing. Ultimately, the cut‐offs defined for the PFA reflect a trade‐off between sensitivity and specificity, with too low a cut‐off potentially capturing false positives, and too high a cut‐off potentially missing true positives.

## 5 CONCLUSIONS

We essentially harmonized NRRs and interpreted comments for PFA testing in our large pathology network, NSWHP, representing the largest public pathology organization in Australia. Harmonization has a number of advantages, as partly explored elsewhere.^55^ We propose our evaluation will be useful for other organizations wishing to harmonize PFA testing in their networks. Moreover, we propose that our NRRs may provide a useful comparator for individual laboratories that perform such testing. In particular, NRRs that greatly differ from those shown here may yield relatively poor performance of PFA for its intended purpose, as a screen of primary haemostasis including VWD. We also propose that the C/ADP CT NRRs recommended by the manufacturer should be revised in line with our data and also that of the vast majority of studies reported in the literature, which show wider ranges with higher upper limit values. Finally, we suspect that the narrow C/ADP ranges provided by the manufacturer, with cut‐off of 100 s, will likely over‐call PFA CTs as “abnormal,” with a cut‐off closer to 120 s likely showing better utility.

## CONFLICT OF INTEREST

The authors have no conflicts of interest.

## Supporting information


**Supplementary Table S1** Study sites participating in this evaluation*
**Supplementary Table S2**. Summary of normality tests “passed” using reference range (NRR) data from current report.*

**Supplementary Figure S1** Study summary. A summary of the main evaluations undertaken as part of this study.

**Supplementary Figure S2** Comparison of closure time (CT) data obtained using PFA‐100 versus PFA‐200 at Site A. Figure A shows data for C/Epi (*n* = 168 samples), and B shows data for C/ADP (*n* = 153 samples), co‐tested over a period of 5 years. Data shown as Box and Whiskers with 10‐90th percentiles. There was no statistically significant difference in test results (*p* values shown, generated using the two‐tailed Mann–Whitney test).

**Supplementary Figure S3** A, B. Comparison of CTs (C/Epi [A] and C/ADP [B]; sec) and platelet count (x10^9^/L) and haematocrit values for normal individuals where combined testing was done. Data from 3 participant laboratory sites. Several high‐outlier CTs obtained for C/Epi shown on right portion of figure; these outliers could not be generally linked to low platelet counts or low haematocrit. C, D. Comparison of CTs (C/Epi [C] and C/ADP [D]; sec) and VWF level (VWF:Ag) and activity (VWF:CB or VWF:RCo) where combined testing was done. Data from 1 participant laboratory site. Several high‐outlier CTs obtained for C/Epi shown on right of figure; these outliers could not be generally linked to low levels of VWF or VWF activity. However, there was a statistically significant relationship between C/Epi and C/ADP (all figures), as well as between C/ADP and VWF level and activity (Figure D), which was not evident for any other comparison. Low cut‐off values for other parameters are as follows: platelet count (150 × 10^9^/L), haematocrit (females; 0.355), VWF level (VWF:Ag, 50 U/dL) or activity (50 U/dL VWF:CB; 40 U/dL VWF:RCo).

## Data Availability

The data that support the findings of this study are available from the corresponding author upon reasonable request.
