# Supplementary material for: Harmonizing platelet function analyzer testing and reporting in a large laboratory network
Source: Int J Lab Hematol. 2022 Jun 26;44(5):934–44. doi: 10.1111/ijlh.13907 (PMC9545980; doi:10.1111/ijlh.13907)
Supplement: Supplementary file 1 — Supplementary Table S1 Study sites participating in this evaluation* Supplementary Table S2. Summary of normality tests “passed” using reference range (NRR) data from current report.* [file IJLH-44-934-s002.docx]

**Supplementary Table 1:** Study sites participating in this evaluation*

| **Study site (NSW Health Pathology)** | **Lab code** | **Instruments available (PFA-)** |
| --- | --- | --- |
| Institute of Clinical Pathology and Medical Research (ICPMR), located at Westmead Hospital, NSW. | A | 100 and 200 |
| John Hunter Hospital (JHH), Newcastle, NSW. | B | 200 |
| Royal North Shore Hospital, St Leonards, NSW Australia | C | 200 |
| Royal Prince Alfred Hospital, Camperdown, NSW. | D | 100 |
| Liverpool Hospital, Liverpool | E | 100 |
| Wollongong Hospital, Wollongong | F | 200 |

* All sites located in NSW, Australia. All sites provided PFA closure time (CT) testing data for this evaluation, using the PFA instruments identified. All PFA tests were performed as per manufacturer guidance, within 4 hours of blood collection. For numbers of samples assessed at each site for each evaluation, please refer to Table 1 and each respective figure in Results. Two additional smaller laboratory sites in our pathology network have a PFA instrument on site, but were not able to provide data for this evaluation.

**Supplementary Table 2.** Summary of normality tests ‘passed’ using reference range (NRR) data from current report.*

| ***Site*** | **ICPMR (A)** | | **JHH/RNSH (B/C)** | | **RPA (D)** | | **Liv (E)** | | **Woll (F)** | | **All (A-F)** | |
| --- | --- | --- | --- | --- | --- | --- | --- | --- | --- | --- | --- | --- |
|  | ***C/Epi*** | ***C/ADP*** | ***C/Epi*** | ***C/ADP*** | ***C/Epi*** | ***C/ADP*** | ***C/Epi*** | ***C/ADP*** | ***C/Epi*** | ***C/ADP*** | ***C/Epi*** | ***C/ADP*** |
| **Number of data points** | 47 | 48 | 46 | 46 | 38 | 42 | 33 | 38 | 20 | 22 | 180 | 194 |
|  |  |  |  |  |  |  |  |  |  |  |  |  |
| ***Normality test*** |  |  |  |  |  |  |  |  |  |  |  |  |
| Anderson-Darling test | Yes | Yes | Yes | No | Yes | Yes | No | No | Yes | Yes | Yes | No |
| D'Agostino & Pearson test | Yes | Yes | Yes | No | Yes | Yes | Yes | Yes | Yes | Yes | Yes | No |
| Shapiro-Wilk test | Yes | Yes | Yes | No | Yes | Yes | No | No | Yes | Yes | Yes | No |
| Kolmogorov-Smirnov test | Yes | Yes | Yes | Yes | Yes | Yes | Yes | No | Yes | Yes | Yes | No |
|  |  |  |  |  |  |  |  |  |  |  |  |  |
| **Summary – passed:** | 4/4 | 4/4 | 4/4 | 1/4 | 4/4 | 4/4 | 2/4 | 1/4 | 4/4 | 4/4 | 4/4 | 0/4 |

*For data shown in Figure 1 (with outliers removed)
