# Supplementary figures and images for: Harmonizing platelet function analyzer testing and reporting in a large laboratory network
Source: Int J Lab Hematol. 2022 Jun 26;44(5):934–44. doi: 10.1111/ijlh.13907 (PMC9545980; doi:10.1111/ijlh.13907)

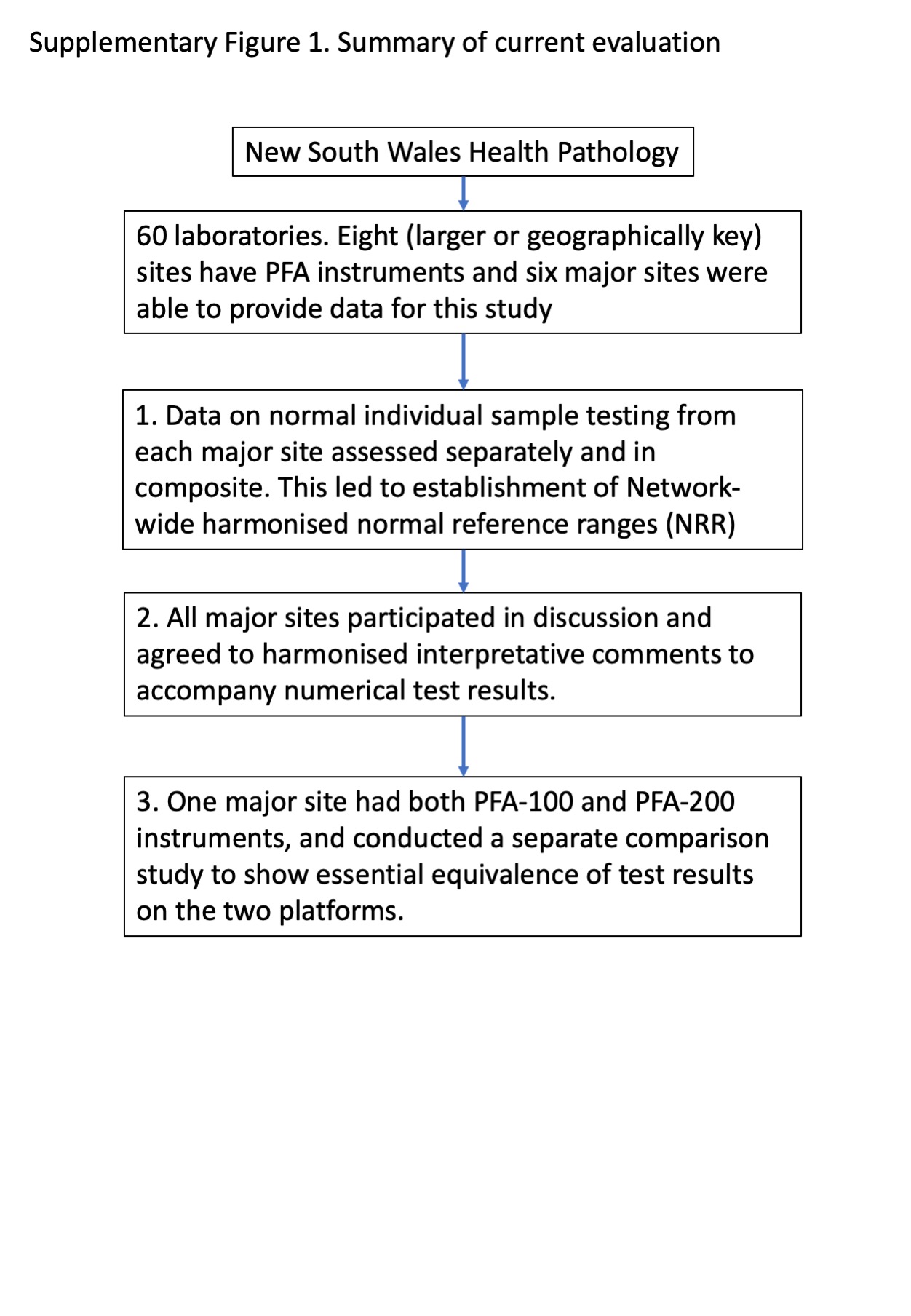

Supplement: Supplementary file 2 — Supplementary Figure S1 Study summary. A summary of the main evaluations undertaken as part of this study. [file IJLH-44-934-s003.jpg]

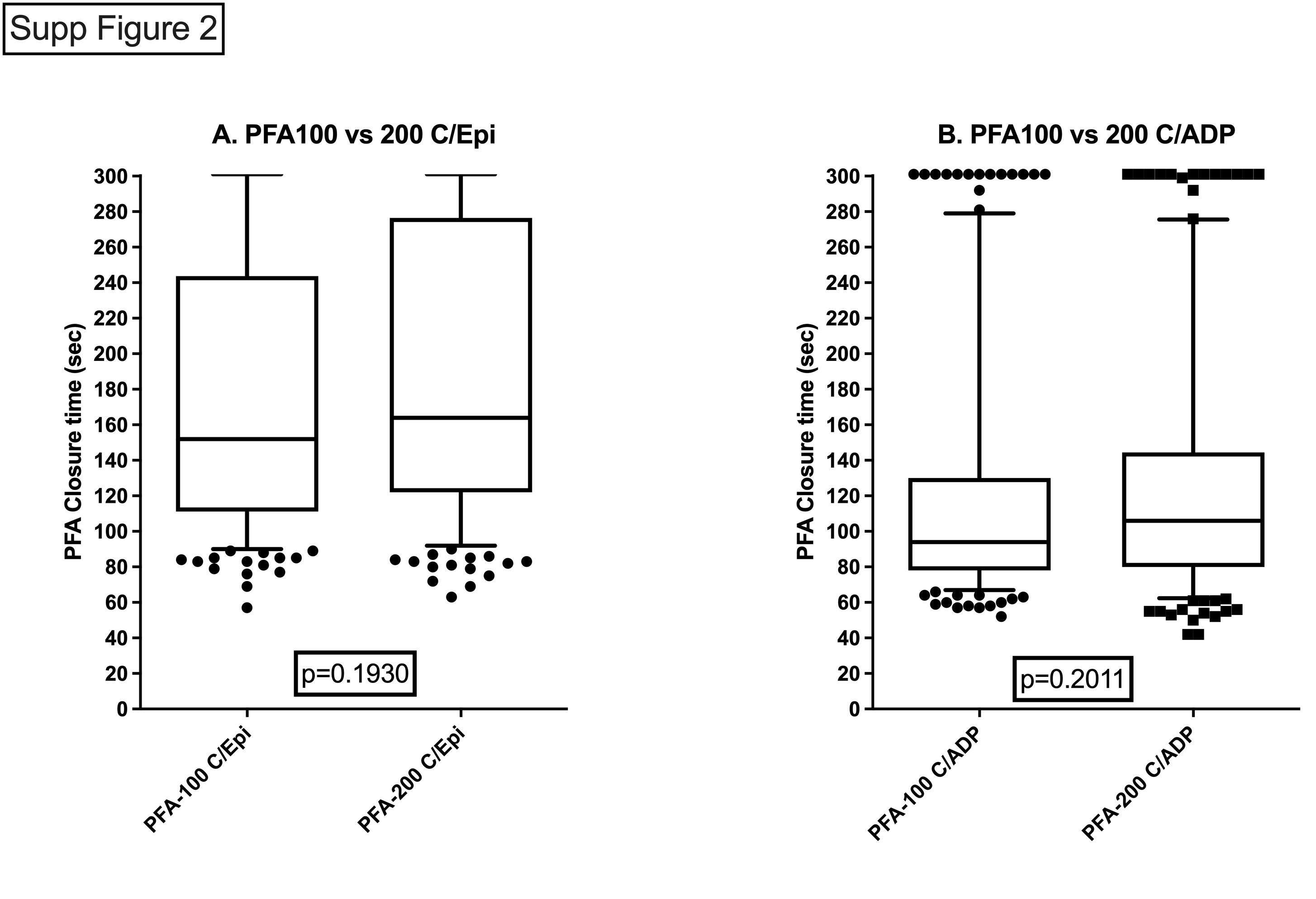

Supplement: Supplementary file 3 — Supplementary Figure S2 Comparison of closure time (CT) data obtained using PFA‐100 versus PFA‐200 at Site A. Figure A shows data for C/Epi (n = 168 samples), and B shows data for C/ADP (n = 153 samples), co‐tested over a period of 5 years. Data shown as Box and Whiskers with 10‐90th percentiles. There was no statistically significant difference in test results (p values shown, generated using the two‐tailed Mann–Whitney test). [file IJLH-44-934-s001.jpg]

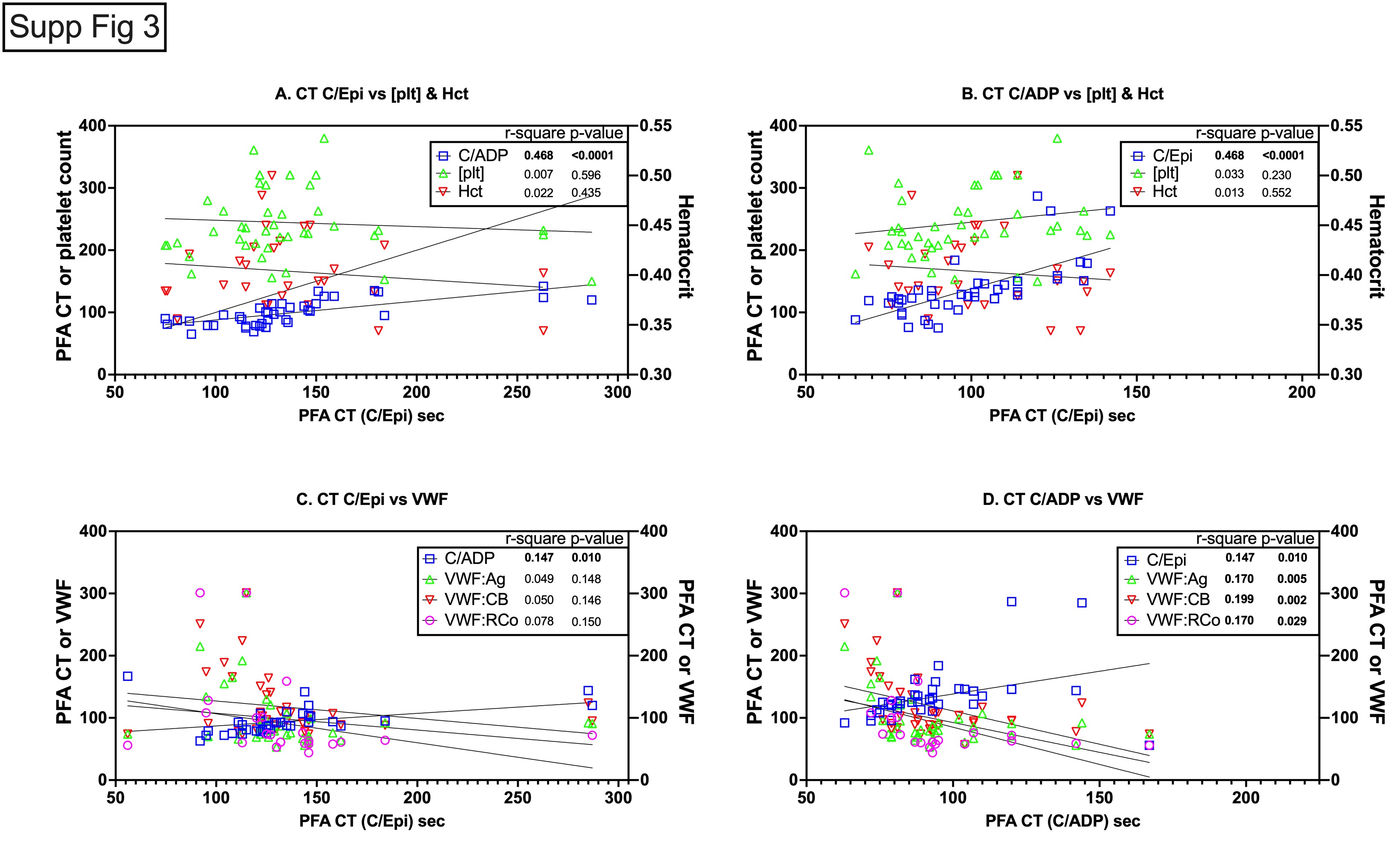

Supplement: Supplementary file 4 — Supplementary Figure S3 A, B. Comparison of CTs (C/Epi [A] and C/ADP [B]; sec) and platelet count (x109/L) and haematocrit values for normal individuals where combined testing was done. Data from 3 participant laboratory sites. Several high‐outlier CTs obtained for C/Epi shown on right portion of figure; these outliers could not be generally linked to low platelet counts or low haematocrit. C, D. Comparison of CTs (C/Epi [C] and C/ADP [D]; sec) and VWF level (VWF:Ag) and activity (VWF:CB or VWF:RCo) where combined testing was done. Data from 1 participant laboratory site. Several high‐outlier CTs obtained for C/Epi shown on right of figure; these outliers could not be generally linked to low levels of VWF or VWF activity. However, there was a statistically significant relationship between C/Epi and C/ADP (all figures), as well as between C/ADP and VWF level and activity (Figure D), which was not evident for any other comparison. Low cut‐off values for other parameters are as follows: platelet count (150 × 109/L), haematocrit (females; 0.355), VWF level (VWF:Ag, 50 U/dL) or activity (50 U/dL VWF:CB; 40 U/dL VWF:RCo). [file IJLH-44-934-s004.jpg]
